# Supplementary material for: Late Blight Resistance Conferred by Rpi-Smira2/R8 in Potato Genotypes In Vitro Depends on the Genetic Background
Source: Plants (Basel). 2022 May 16;11(10):1319. doi: 10.3390/plants11101319 (PMC9145795; doi:10.3390/plants11101319)
Supplement: Supplementary file 1 [file plants-11-01319-s001.zip › plants-1713452-supplementary.pdf]

**Supplementary information**

Manuscript

# Late blight resistance conferred by *Rpi-Smira2/R8* in potato genotypes *in vitro* depends on the genetic background

**Authors:** Eva Blatnik[1], Marinka Horvat[2], Sabina Berne[2], Miha Humar[3], Peter Dolničar[1], Vladimir Meglič[1]

[1] Crop Science Department, Agricultural Institute of Slovenia, Hacquetova ulica 17, SI-1000 Ljubljana, Slovenia

[2] Department of Agronomy, Biotechnical Faculty, University of Ljubljana, Jamnikarjeva ulica 101, SI-1000 Ljubljana, Slovenia

[3] Department of Wood Science and Technology, Biotechnical Faculty, University of Ljubljana, Jamnikarjeva ulica 101, SI-1000 Ljubljana, Slovenia

**Corresponding author:** Eva Blatnik,

eva.blatnik@kis.si

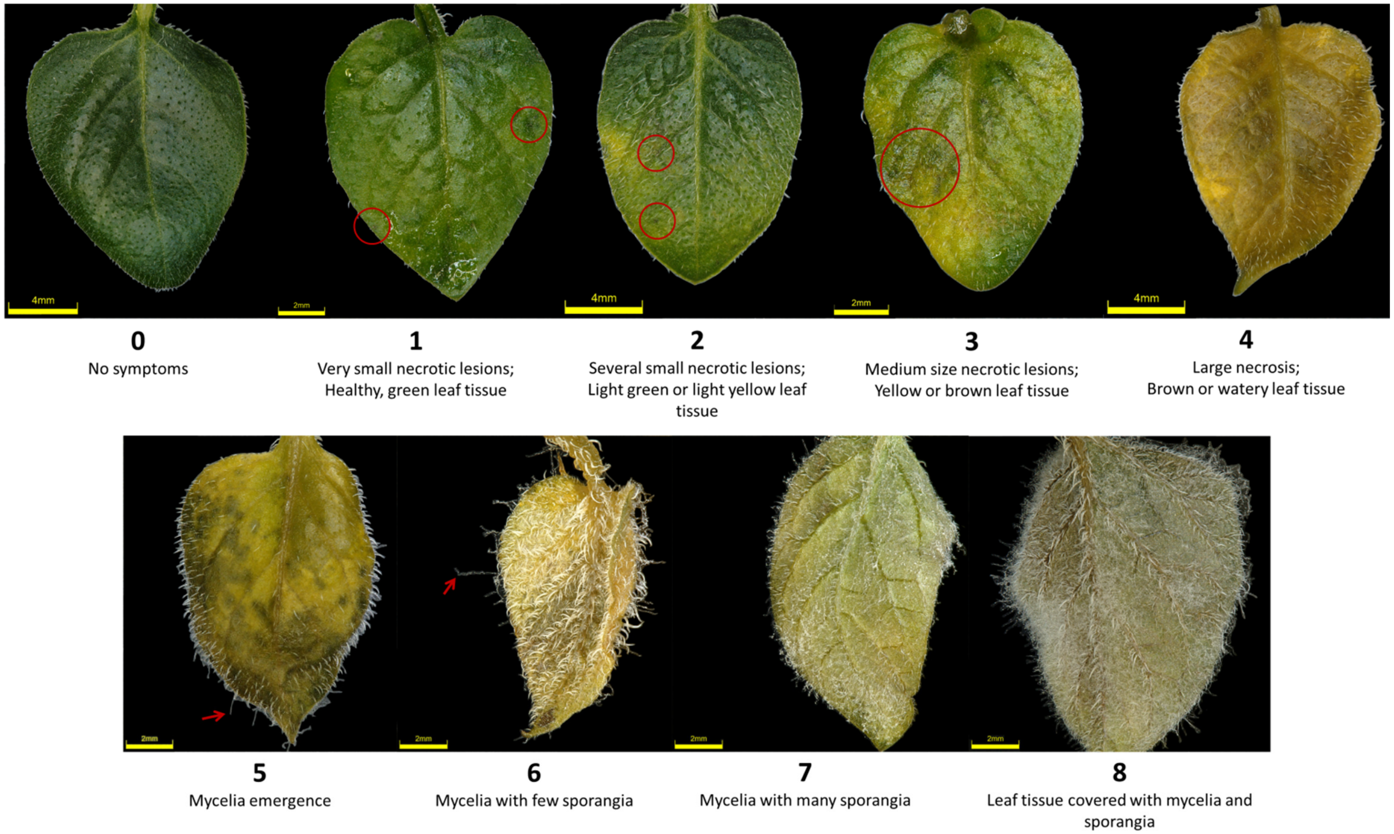


**Supplementary Figure S1: Disease rating scale used for determining the severity of late blight after inoculation of *R8* genotypes with *P. infestans* with representative leaflets for each disease score.** Necrotic lesions are indicated with red circles, while mycelia and sporangia are marked with red arrows. All images were taken with darkfield illumination and 20x magnification.

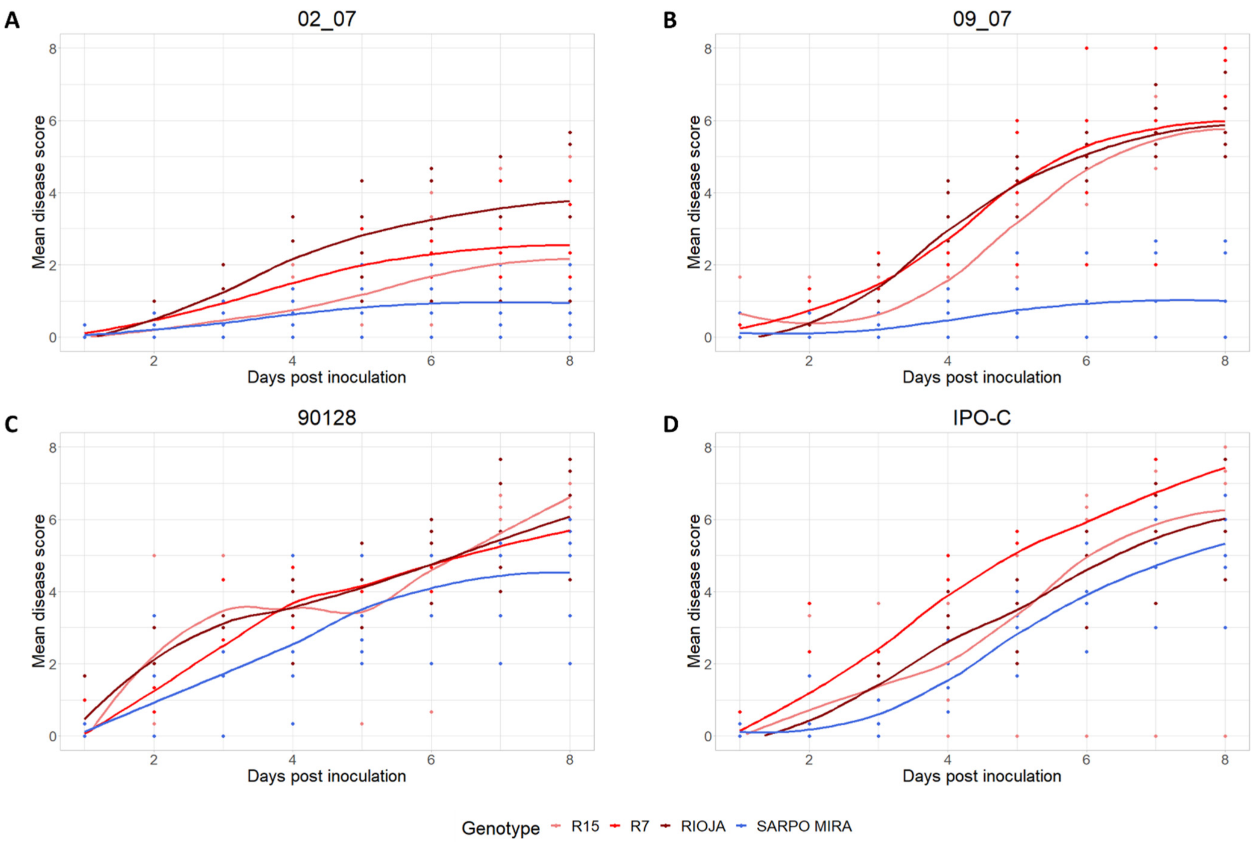


**Supplementary Figure S2: Late blight disease progression curves of the Rioja cross group show differences in resistance levels between progeny *R8* genotypes and parental potato cultivars after inoculation with isolate 02_07 (A), isolate 09_07 (B), isolate 90128 (C) and isolate IPO-C (D).** Both progeny *R8* genotypes and parental cultivars were resistant to isolate 02_07. After inoculation with isolates 09_07, 90128 and IPO-C the genotypes R7 and R15 had similar disease response to Rioja, and were all either intermediately resistant or susceptible.

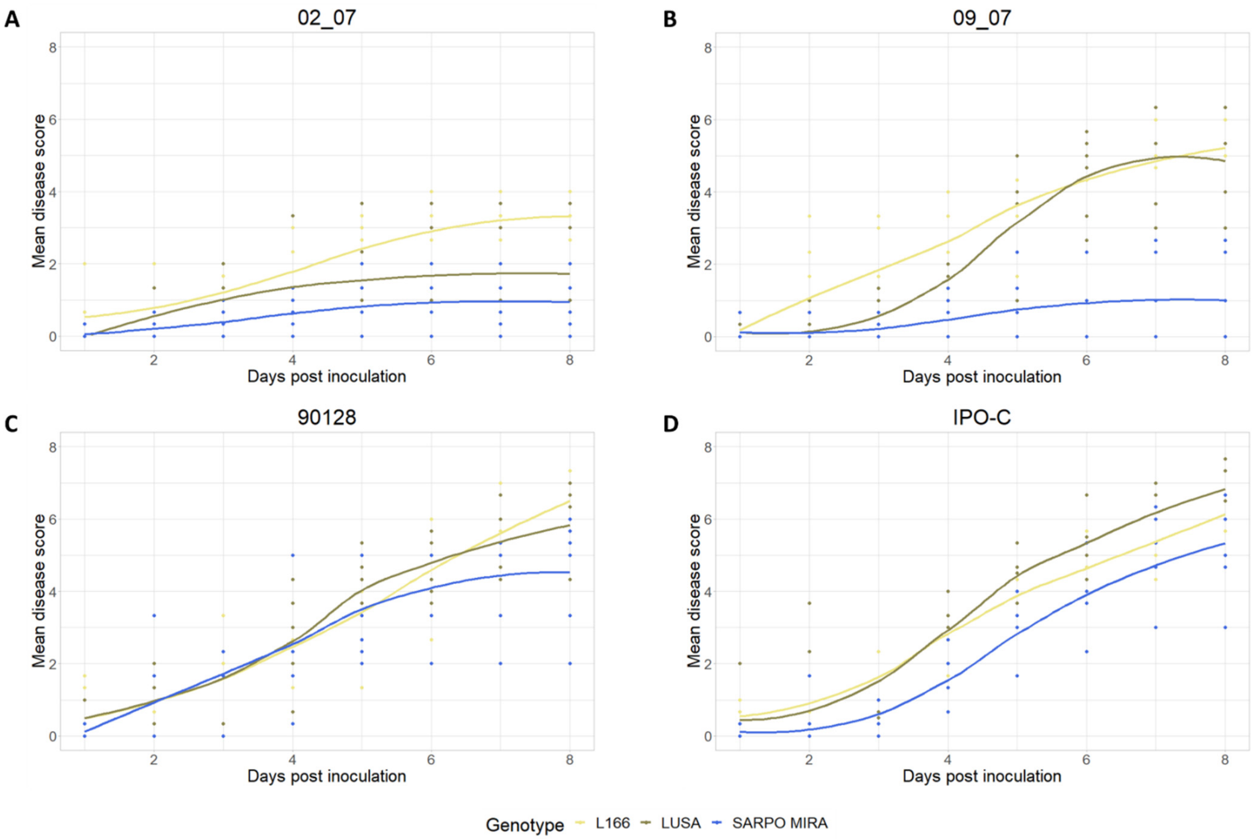


**Supplementary Figure S3: Late blight disease progression curves of the Lusa cross group show differences in resistance levels between progeny *R8* genotypes and parental potato cultivars after inoculation with isolate 02_07 (A), isolate 09_07 (B), isolate 90128 (C) and isolate IPO-C (D).** Genotype L166 was the only progeny genotype that showed more severe symptoms compared to the susceptible parental cultivar Lusa.

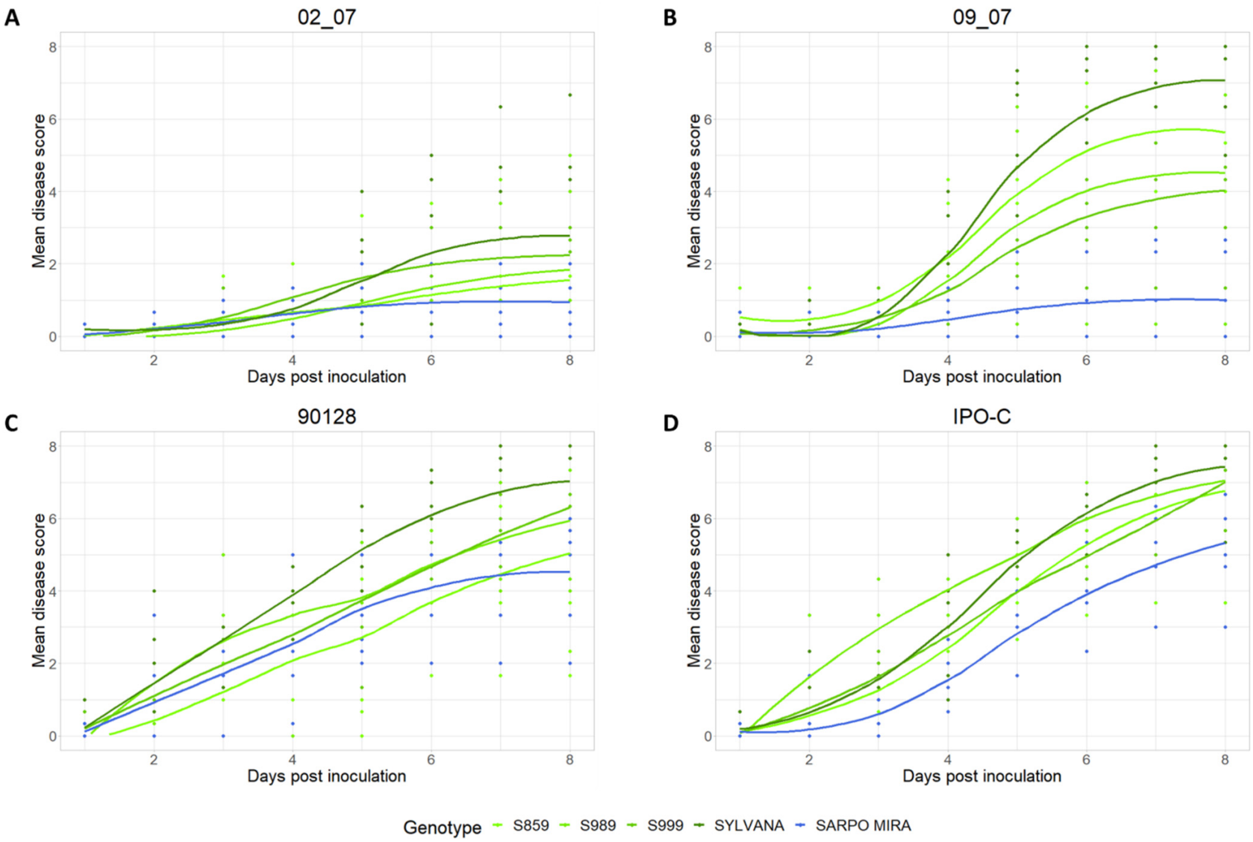


**Supplementary Figure S4: Late blight disease progression curves of the Sylvana cross group show differences in resistance levels between progeny *R8* genotypes and parental potato varieties after inoculation with isolate 02_07 (A), isolate 09_07 (B), isolate 90128 (C) and isolate IPO-C (D).** Genotypes S989 and S999 showed milder symptoms compared to the susceptible parental cultivar Sylvana only in the case of isolate 09_07.
